# A subcentimeter duodenal neuroendocrine neoplasm with a liver metastasis upgraded to G3: a case report

**DOI:** 10.1186/s40792-021-01155-1

**Published:** 2021-03-19

**Authors:** Tomoya Kitada, Toshihiko Masui, Yosuke Kasai, Yuichiro Uchida, Satoshi Ogiso, Takashi Ito, Takamichi Ishii, Satoru Seo, Hiroyuki Katsuragawa, Shinji Uemoto

**Affiliations:** 1 Department of Surgery, Graduate School of Medicine, Kyoto University, 54 Kawahara-cho, Shogoin, Sakyo-ku, Kyoto, 606-8507 Japan; 2 Department of Diagnostic Pathology, Graduate School of Medicine, Kyoto University, 54 Kawahara-cho, Shogoin, Sakyo-ku, Kyoto, 606-8507 Japan

**Keywords:** Duodenal neuroendocrine neoplasm, Intratumor heterogeneity, High grade, Liver metastasis

## Abstract

**Background:**

Although duodenal neuroendocrine neoplasms (DuNENs) usually have indolent phenotypes, some DuNENs exhibit aggressive clinical manifestations. Tumor size > 1 cm, lymph node metastasis, and high grade have been associated with poor prognosis. However, preoperative risk evaluation is often difficult, because Ki-67 index on biopsy is frequently underestimated due to the intratumor heterogeneity. Here, we present a case of a subcentimeter DuNEN with a low Ki-67 index on endoscopic biopsy, who developed lymph node metastasis and high-grade liver metastasis.

**Case presentation:**

The patient was a 52-year-old female who presented an epigastric pain. Esophagogastroduodenoscopy revealed a duodenal submucosal lesion with a size of 8 mm. The endoscopic biopsy showed DuNEN with a Ki-67 index of 3.3% (G2 categorized by the World Health Organization 2019 classification). We performed an open partial duodenectomy with adjacent lymph node dissection. Pathological examination of the resected specimens revealed a Ki-67 index of 13.5% (G2) in the “hot spot” and lymph node metastasis. A hepatic low-density area detected on preoperative contrast-enhanced computed tomography appeared to be a liver metastasis on postoperative gadoxetic acid-enhanced magnetic resonance imaging. Subsequently, we performed a laparoscopic partial hepatectomy. Pathological examination of the liver specimen showed a metastatic neuroendocrine tumor with a Ki-67 index of 27.5% (NET-G3). The patient has been alive for 14 months since the hepatectomy.

**Conclusions:**

This case shows the possibility of high malignant potential of DuNEN even if the primary lesion is < 1 cm and has a low Ki-67 index on biopsy.

## Background

Neuroendocrine neoplasm (NEN) is a rare disease with an age-adjusted annual incidence of 6.98 per 100,000 population in the United States [1]. Duodenal NEN (DuNEN) accounts for 2.8% of NENs [2]. Although DuNENs usually have indolent phenotypes, some clinicopathological features, including tumor size > 1 cm, lymph node metastasis, and high grade, have been associated with poor prognosis [3, 4]. However, preoperative risk evaluation is often difficult, because Ki-67 index on biopsy of NENs is frequently underestimated due to the intratumor heterogeneity [5, 6]. Here, we present a case of a subcentimeter DuNEN with a low Ki-67 index on endoscopic biopsy, who developed lymph node metastasis and high-grade liver metastasis.

## Case presentation

**Fig. 1:**
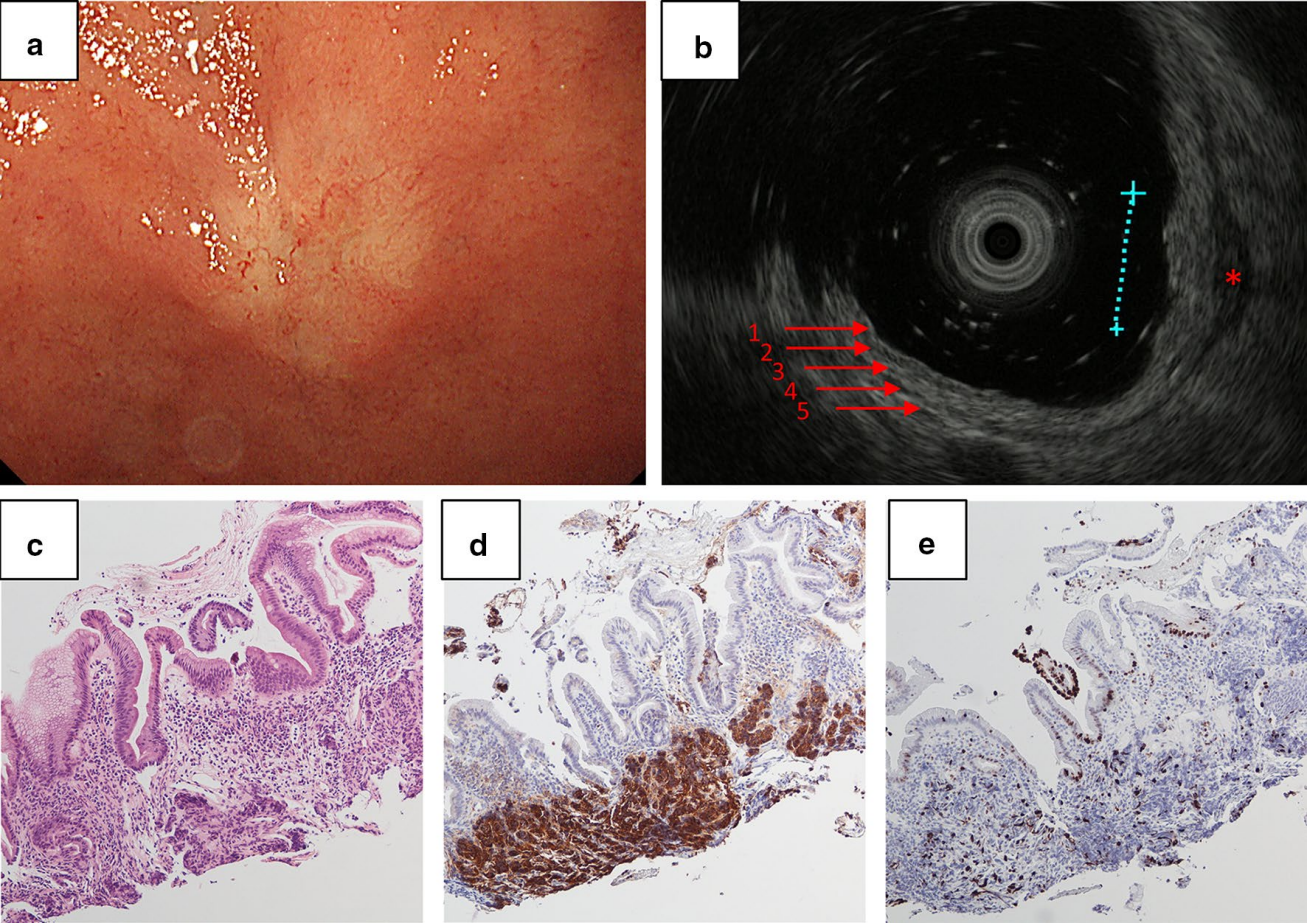
**a** Esophagogastroduodenoscopy showing a submucosal lesion in the duodenal bulb. **b** Endoscopic ultrasound showing a submucosal lesion with 8 mm in size. The numbers (1–5) indicate the order of the layers. *Thickened fourth layer beneath the lesion. **c** Hematoxylin and eosin staining and immunohistochemistry for **d** chromogranin A and **e** Ki-67 of the biopsy specimen

**Fig. 2:**
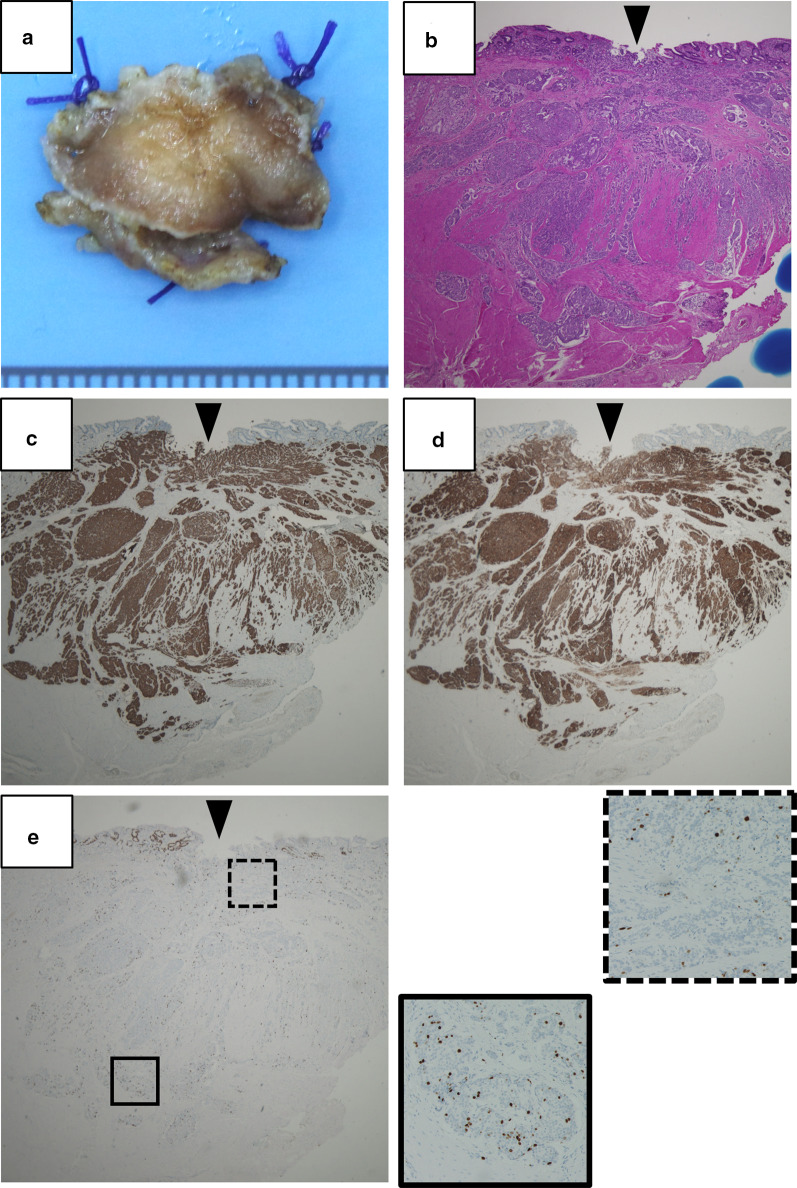
Gross appearance and microscopic findings of the primary DuNEN. **a** Duodenal specimen (mucosal side). **b** Hematoxylin and eosin staining and immunohistochemistry for **c** chromogranin A, **d** somatostatin receptor type 2 (SSTR2), and **e** Ki-67. Arrowheads indicate the site of biopsy. The small panels framed by straight and dotted lines of **e** are × 4 magnified from each area indicated in the large panel. The original magnification of large panels is × 20

The patient was a 52-year-old female who presented an epigastric pain persisting for 6 months. Esophagogastroduodenoscopy showed a small submucosal lesion in the anterior wall of the duodenal bulb (Fig. 1a). Endoscopic ultrasound (EUS) revealed an ill-circumscribed lesion with the main focus in the third layer of the duodenum (the submucosal layer, Fig. 1b). The estimated size was 8 mm. The fourth layer (the layer of the muscularis propria) beneath the lesion was focally thickened, suggesting slight invasion of the muscularis propria. Endoscopic biopsy was performed, and the patient was diagnosed as DuNEN with positive chromogranin A and a Ki-67 index of 3.3% [G2 categorized by the World Health Organization (WHO) 2019 classification [7], Fig. 1c–e]. There was a small low-density area in the segment 5 of the liver on contrast-enhanced computed tomography (CE-CT) with unclear significance. Because of the small size and marginal G1/G2 of the primary tumor, we did not emphasize its significance. On multidisciplinary discussion, endoscopic resection was not indicated due to the possible invasion of muscularis propria; pancreaticoduodenectomy was considered to be too invasive a procedure for the small size and tumor location. Hence, we decided to perform a partial duodenectomy. On laparotomy, there were no apparent findings of liver metastasis or peritoneal dissemination. The tumor was located in the anterior wall of the duodenal bulb, and the whole layer resection of the duodenum was performed. The duodenal wall was closed by the Albert–Lembert suture in the transverse direction. Adjacent lymph nodes were dissected (#5, #8a, #12p, and #13 according to the Classification of Pancreatic Carcinoma from Japan Pancreas Society [8]). The patient recovered well without complications and was discharged on postoperative day 14. Pathological examination of the resected specimens revealed DuNEN with positive chromogranin A and somatostatin receptor type 2 (SSTR2, Fig. 2a–d). The tumor exhibited an infiltrative pattern extensively involving the muscularis propria (Fig. 2b). The site of biopsy was observed as an erosion at the very superficial part of the entire lesion. Nuclear staining of Ki-67 was heterogenous across the lesion; relatively higher in the invasive front than in the superficial part near the site of biopsy (Fig. 2e). Ki-67 index was calculated as 13.5% (G2) in the “hot spot”. Lymph node metastasis was present in 1 of 11 nodes examined [a node in the anterosuperior group along the common hepatic artery (#8a)].

**Fig. 3:**
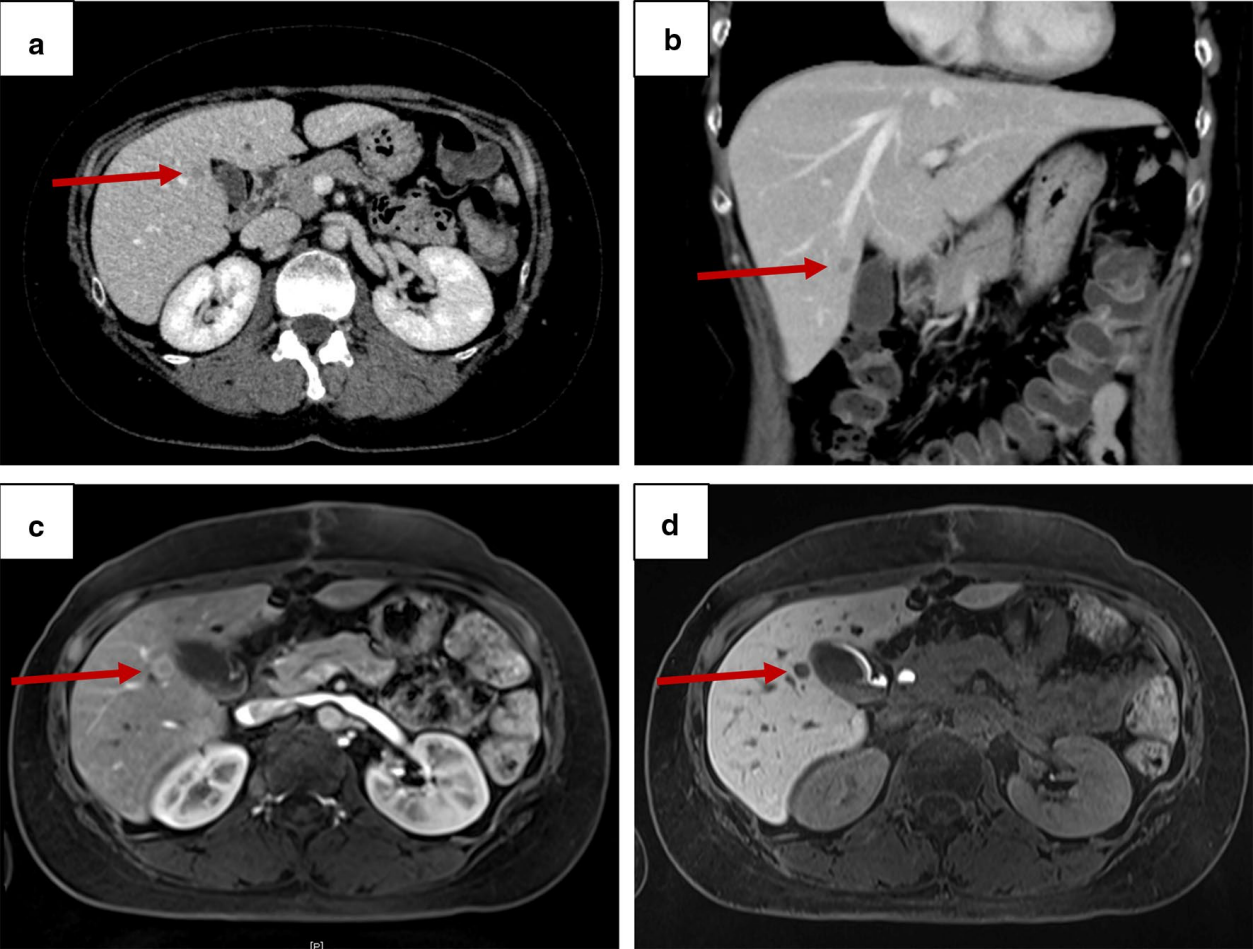
**a**, **b** Contrast-enhanced computed tomography showing a low-density area in the segment 5 of the liver. **a** Axial, and **b** coronal images. **c**, **d** Gadoxetic acid-enhanced magnetic resonance imaging showing a solitary **c** hyperenhancing lesion on arterial phase and **d** defect on hepatocyte phase. Arrows indicate the lesion

**Fig. 4:**
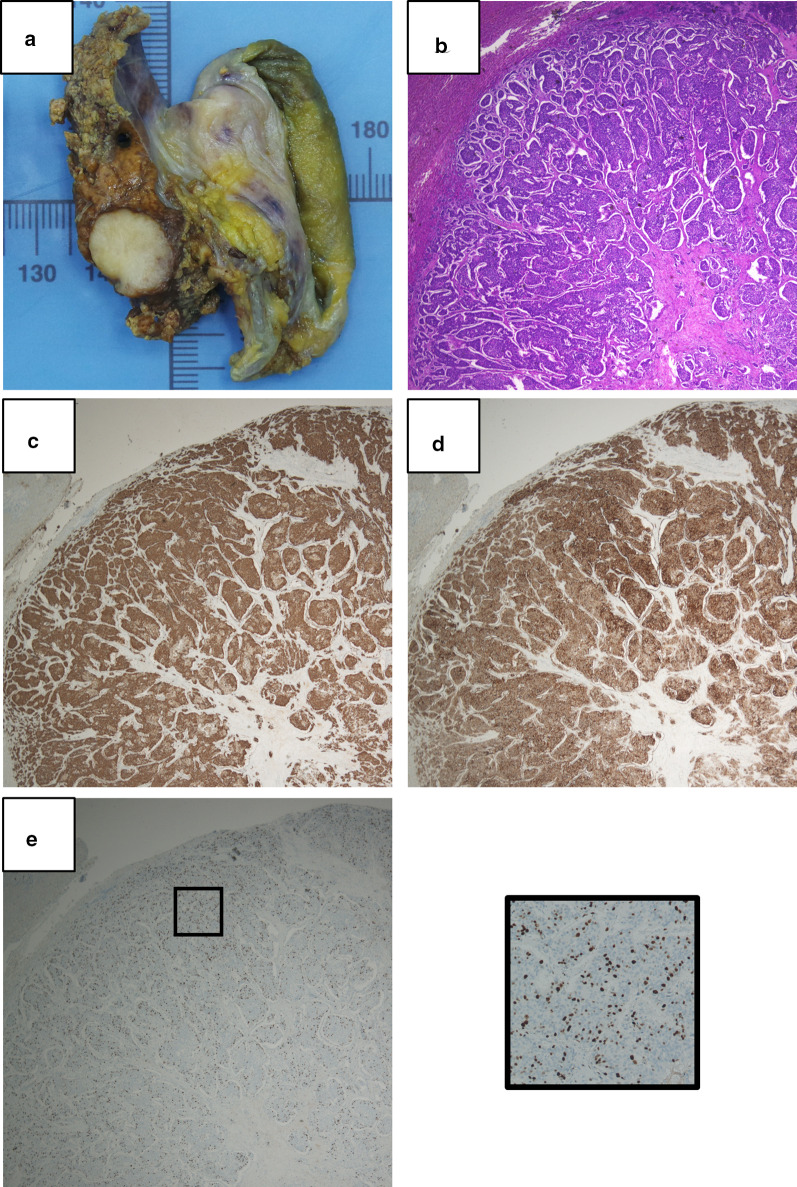
Gross appearance and microscopic findings of the liver metastasis. **a** The representative section of the liver specimen. **b** Hematoxylin and eosin staining and immunohistochemistry for **c** chromogranin A, **d** somatostatin receptor type 2 (SSTR2), and **e** Ki-67. The panel of **e** is × 4 magnified from the area indicated in **e**. The original magnification of panels is × 20

Because the malignant potential was higher than expected on preoperative workup, the hepatic low-density area detected on preoperative CE-CT (Fig. 3a, b) was elucidated by gadoxetic acid-enhanced magnetic resonance imaging and appeared to be a solitary liver metastasis with 8 mm in size (Fig. 3c, d). Two months after the initial surgery, the patient underwent a laparoscopic partial hepatectomy of the segment 5. The postoperative course was uneventful, and the patient was discharged on postoperative day 14. Pathological examination of the liver specimen showed a metastatic neuroendocrine tumor (NET) with well-differentiated morphology and positive chromogranin A and SSTR2 (Fig. 4a–d). Notably, nuclear staining of Ki-67 was much conspicuous in the liver metastasis compared to the primary DuNEN, and the Ki-67 index was calculated as 27.5% (NET-G3, Fig. 4e).

**Fig. 5:**
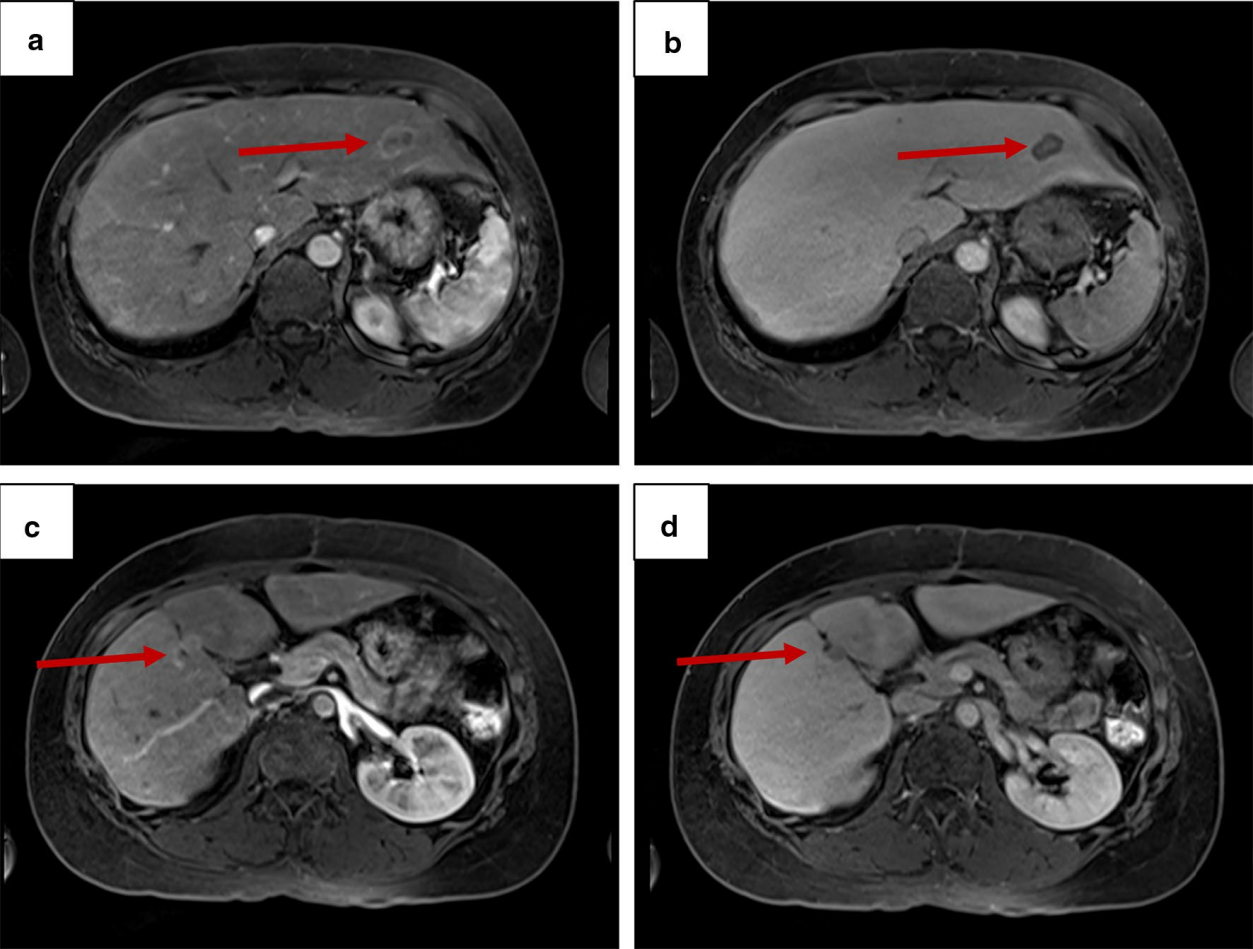
Recurrence in the liver 1 year after the hepatectomy. Gadoxetic acid-enhanced magnetic resonance imaging showing two liver lesions in the segment 2 (**a** and **b**) and segment 4/5 (**c** and **d**) with hyperenhancement on arterial phase (**a** and **c**) and defect on hepatocyte phase (**b** and **d**). Arrows indicate the lesions

The patient was observed without adjuvant therapy, but multiple liver metastases appeared 1 year after the hepatectomy (Fig. 5a–d). Thereafter, she has been managed by medical treatments with everolimus and lanreotide. She has been alive for 14 months since the hepatectomy.

## Discussion

Although DuNENs usually have indolent phenotypes, tumor size > 1 cm, invasion of the muscularis propria, lymph node metastasis, and high grade have been associated with poor prognosis [3, 4, 9]. The present case had a primary tumor < 1 cm, no radiological evidence of lymph node metastasis, and marginal *G*1/*G*2 on preoperative biopsy, whereas the finding of EUS suggested slight invasion of the muscularis propria. Collectively, these preoperative findings did not strongly suggest high malignant potential. Hence, we did not emphasize the significance of the hepatic low-density area appreciated on preoperative CE-CT, and did not examine the patient by somatostatin receptor scintigraphy. However, the pathology showed that the primary DuNEN actually exhibited an infiltrative pattern extensively involving the muscularis propria, as shown in Fig. 2. Retrospectively, the microscopic finding coincided well with the finding of the thickened layer of the muscularis propria on EUS (Fig. 1b). In addition, there was an intratumor heterogeneity of nuclear staining of Ki-67, where the expression was higher in the deep invasive front than the superficial part (Fig. 2e). In general, Ki-67 index tends to be underestimated on EUS-fine needle aspiration (FNA) due to the intratumor heterogeneity in NENs [5, 6]. The present case had a submucosal lesion which was directly accessible by endoscopy and larger tissue was available for histologic evaluation than that obtained by EUS-FNA. Nevertheless, only the very superficial part of the entire lesion was biopsied, as shown in Fig. 2e. These would be the reason for the discrepancy of Ki-67 indices between the endoscopic biopsy and the surgical specimen of the primary DuNEN.

Notably, the present case exhibited a grade increase to G3 in the liver metastasis. This might be the result that the high-proliferative cells in the invasive front of the primary DuNEN metastasized to the liver. In a retrospective study from Italy including 108 DuNENs, G3 accounted for 4% and was associated with 30-fold increased risk of death and progression compared to G1 [3]. In the WHO 2019 classification, gastrointestinal NENs-G3 were reclassified as well-differentiated NET-G3 and poorly differentiated neuroendocrine carcinoma (NEC) [7]. NET-G3 behave relatively benignly compared to NEC [10–12]. The National Comprehensive Cancer Network guidelines group NET-G3 together with NET-G1/G2, where metastasectomy is recommended if complete resection is possible [13]. In the present case, both the primary tumor and liver metastasis showed well-differentiated morphology and expressed typical neuroendocrine markers (chromogranin A and SSTR2), which indicated the liver metastasis to be well-differentiated NET-G3. Although we had not confirmed the diagnosis of the liver metastasis as NET-G3 preoperatively, resection of the solitary liver metastasis should be appropriate in accordance with the guidelines’ recommendation.

We extensively searched previous case reports on subcentimeter primary gastrointestinal NET-G1/G2 which upgraded to G3 in the liver metastasis, and found one such case of rectal origin [14]. In that case, an 8-mm rectal NET-G1 was endoscopically resected completely, which recurred in the liver (G3) 5 years later. To our knowledge, ours is the first case report of duodenal origin.

## Conclusions

Although the present case showed a subcentimeter, marginal G1/G2 DuNEN on preoperative workup, the tumor did exhibit extensive infiltration and high proliferative activity in the invasive front and was upgraded to G3 in the liver metastasis. The present case alerts to the possible malignant potential of DuNEN even if the preoperative workup shows benign findings.

## Data Availability

The data supporting the conclusions of this case report are included within the article.

## Abbreviations

CE-CT: Contrast-enhanced computed tomography
DuNEN: Duodenal neuroendocrine neoplasm
EUS: Endoscopic ultrasound
FNA: Fine needle aspiration
NEC: Neuroendocrine carcinoma
NEN: Neuroendocrine neoplasm
NET: Neuroendocrine tumor
SSTR2: Somatostatin receptor type 2
WHO: World Health Organization

## References

[1] Dasari A, Shen C, Halperin D (2017). Trends in the incidence, prevalence, and survival outcomes in patients with neuroendocrine tumors in the United States. JAMA Oncol.

[2] Modlin IM, Lye KD, Kidd M (2003). A 5-decade analysis of 13,715 carcinoid tumors. Cancer.

[3] Massironi S, Campana D, Partelli S (2018). Heterogeneity of duodenal neuroendocrine tumors: an Italian multi-center experience. Ann Surg Oncol.

[4] Masui T, Sato A, Nakano K (2018). Comparison of recurrence between pancreatic and duodenal neuroendocrine neoplasms after curative resection: a single-institution analysis. Ann Surg Oncol.

[5] Yang Z, Tang LH, Klimstra DS (2011). Effect of tumor heterogeneity on the assessment of Ki67 labeling index in well-differentiated neuroendocrine tumors metastatic to the liver: implications for prognostic stratification. Am J Surg Pathol.

[6] Boutsen L, Jouret-Mourin A, Borbath I (2018). Accuracy of pancreatic neuroendocrine tumour grading by endoscopic ultrasound-guided fine needle aspiration: analysis of a large cohort and perspectives for improvement. Neuroendocrinology.

[7] Nagtegaal ID, Odze RD, Klimstra D (2020). The 2019 WHO classification of tumours of the digestive system. Histopathology.

[8] Japan Pancreas Society (2017). Classification of pancreatic carcinoma fourth English edition.

[9] Park SG, Lee BE, Kim GH (2019). Risk factors for lymph node metastasis in duodenal neuroendocrine tumors: a retrospective, single-center study. Medicine (Baltimore).

[10] Busico A, Maisonneuve P, Prinzi N (2019). Gastroenteropancreatic high-grade neuroendocrine neoplasms (H-NENs): histology and molecular analysis, two sides of the same coin. Neuroendocrinology.

[11] Sorbye H, Kong G, Grozinsky-Glasberg S (2020). PRRT in high-grade gastroenteropancreatic neuroendocrine neoplasms (WHO G3). Endocr Relat Cancer.

[12] Tang LH, Untch BR, Reidy DL (2016). Well-differentiated neuroendocrine tumors with a morphologically apparent high-grade component: a pathway distinct from poorly differentiated neuroendocrine carcinomas. Clin Cancer Res.

[13] NCCN clinical practice guidelines in oncology: neuroendocrine and adrenal tumors 2.2020. Accessed Dec 2020 ed, 2020.

[14] Jo IH, Lee KM, Kim DB (2020). Low-grade rectal neuroendocrine tumor recurring as multiple hepatic metastasis after complete endoscopic removal: a case report. Korean J Gastroenterol.

